# Imported Human Babesiosis, Singapore, 2018

**DOI:** 10.3201/eid2604.200025

**Published:** 2020-04

**Authors:** Poh-Lian Lim, Jean-Marc Chavatte, Shawn Vasoo, Jonathan Yang

**Affiliations:** Tan Tock Seng Hospital, Singapore, (P.-L. Lim, S. Vasoo);; Nanyang Technological University, Singapore (P.-L. Lim, S. Vasoo);; National Centre for Infectious Diseases, Singapore (J.-M. Chavatte);; Eton College, Windsor, UK (J. Yang)

**Keywords:** Babesiosis, *Babesia microti*, Singapore, United States, travel, tickborne diseases, zoonoses, vector-borne infections, bacteria

## Abstract

In 2018, *Babesia microti* infection was diagnosed for a 37-year-old man in Singapore who acquired the infection in the United States. This case highlights the recent rise of tickborne infections in the United States and the risk for their spread, because of increasing global interconnectivity, to regions where they are not endemic.

*Babesia* spp. are intra-erythrocytic protozoal organisms that can infect mammals and birds. Human babesiosis is an emerging tickborne zoonosis, caused mainly by *Babesia microti* and transmitted by ixodid ticks. It is endemic to the United States (*1**–**3*) and, to a lesser extent, China (*3*,*4*). Recently, sporadic cases of human babesiosis caused by several species of *Babesia* have been reported in other countries: *B. microti* (Germany, Australia, South Korea), *B. microti*–like (Japan, Taiwan, China), *B. duncani* (United States, Canada), *B. divergens* (Europe), *B. venatorum* (Europe, China), *B. crassa*–like (China), *B. motasi*–like (South Korea), and other cases elsewhere (*1**–**4*).

In humans, babesiosis can cause mild influenza-like signs and symptoms, but it can also cause hemolytic anemia and severe infections, especially in asplenic or immunocompromised persons (*1*,*3*). Cases of congenital and transfusion-related transmission have been reported (*1*–*4*). Since 2011–2015, babesiosis incidence in the United States has risen (*2*,*5*). Travel-related tickborne infections in general (*6*) and cases acquired from North America have been reported (*3*,*6*,*7*). To our knowledge, no case of human babesiosis has been reported in Singapore, but cases of *Babesia* infection in canids and birds have been recorded (*8*), suggesting presence of potentially receptive ticks.

On July 23, 2018, a 37-year-old man from the United States sought care at Tan Tock Seng Hospital, Singapore, reporting fever and other influenza-like signs and symptoms that had started on July 5. The patient had resided in Singapore since 2012, working as a finance professional, but he had traveled to multiple places in the year before his illness. In 2017, he vacationed in Vietnam (Ho Chi Minh City, Danang), Thailand (Bangkok, Pattaya), Indonesia (Lombok, Anambas Islands), and Cambodia (Phnom Penh), all without having received pretravel typhoid vaccine or malaria prophylaxis. In 2018, he traveled to Indonesia (Bali) in January and March, then to the United States during June 14–25, where he visited friends and relatives in Boston (MA), Nantucket (MA), and New York (NY).

The patient did not recall any tick bites but on June 17 noticed a right ankle papule, which lasted 3 weeks. He sought consultation at a travel clinic because of high fever (104°F), rigors, and headaches, which had persisted and worsened over 18 days. His fever had not resolved with amoxicillin, which he had started taking a week after symptoms onset. He had no relevant medical history or allergies and was taking no other medication. Physical examination findings were unremarkable, including absence of jaundice, hepatosplenomegaly, or eschars.

Laboratory test results revealed moderate thrombocytopenia and anemia, and malaria blood films revealed trophozoites forming in erythrocytes, suggestive of *Babesia*. The National Public Health Laboratory in Singapore differentiated between malaria and babesiosis by performing microscopy and PCR for both parasites, confirming the presence of *B. microti* and excluding *Plasmodium*. Because of the risk for concomitant tickborne infections, the reference laboratory at the Mayo Clinic (Rochester, MN) conducted PCR testing for *Babesia*, *Ehrlichia*, *Anaplasma*, and *Borrelia burgdorferi* and serologic testing for *Rickettsia rickettsii*. Results confirmed *B. microti* infection and excluded those concomitant tickborne infections. The National Public Health Laboratory further characterized the parasites by using PCR and sequencing according to (*8*) for the *18S ssrRNA* (GenBank accession no. MK609547) and the mitochondrial *cox1* (GenBank accession no. MK609548) genes and genotyping based on the internal transcribed spacer region (GenBank accession no. MK609547), which identified the *Babesia* strain as the type most commonly found in the United States (Figure).

**Figure:**
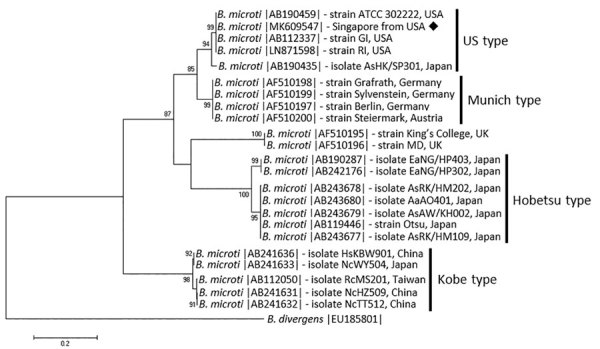
Molecular phylogeny of *Babesia microti* types based on the internal transcribed spacer region. Analysis inferred by maximum likelihood using the general time reversible plus gamma model showing sequence MK609547 from a human patient in Singapore, 2018 (black diamond) and 22 sequences of *B. microti* isolates from diverse geographic areas, retrieved from GenBank (accession numbers provided). Bootstrap values were 10,000 replicates, >85% shown. Scale bar indicates nucleotide substitution rate per site.

After diagnosis, the patient received outpatient treatment with quinine and clindamycin and recovered uneventfully. Consistent with US clinical guidelines, he was advised to not donate blood indefinitely.

This case of travel-acquired human babesiosis diagnosed in Singapore highlights the clinical importance of considering tickborne infections in any traveler with compatible clinical signs and symptoms returning from summertime travel in the United States. It also highlights the challenges of differentiating between malaria and babesiosis in patients who have traveled to areas where both infections are endemic. Even in countries with good access to diagnostic testing, babesiosis may be missed or misdiagnosed as malaria (*7*).

Vectors for babesiosis in canids and bovids have been reported among tick fauna in Singapore (*9*), but to our knowledge, no such vectors for human babesiosis have been reported, which limits the risk for introduction and subsequent autochthonous transmission. However, babesiosis is the most common serious infectious pathogen transmitted by blood transfusions in the United States, and the US Food and Drug Administration has issued screening recommendations to reduce the risk for transfusion-transmitted babesiosis (*10*).

Given the increased incidence of babesiosis and other tickborne bacterial diseases in the United States and the high volumes of international travel, the risk of persons with travel-acquired babesiosis subsequently causing transfusion-transmitted infections is real, albeit small. In many countries, the blood supply is not screened for nonendemic, rare, or geographically limited pathogens, such as *Trypanosoma cruzi* (Chagas disease). Although screening for babesiosis in blood supplies outside the United States may not be financially or logistically feasible, mitigating risk by raising clinician and public health awareness of this emerging problem may be possible.

## References

[1] Vannier E, Krause PJ. Human babesiosis. N Engl J Med. 2012;366:2397–407. 10.1056/NEJMra1202018 22716978

[2] Gray EB, Herwaldt BL. Babesiosis Surveillance - United States, 2011-2015. MMWR Surveill Summ. 2019;68:1–11. 10.15585/mmwr.ss6806a1 31145719

[3] Ord RL, Lobo CA. Human babesiosis: pathogens, prevalence, diagnosis and treatment. Curr Clin Microbiol Rep. 2015;2:173–81. 10.1007/s40588-015-0025-z 26594611 PMC4649939

[4] Chen Z, Li H, Gao X, Bian A, Yan H, Kong D, et al. Human Babesiosis in China: a systematic review. Parasitol Res. 2019;118:1103–12. 10.1007/s00436-019-06250-9 30770979

[5] Paules CI, Marston HD, Bloom ME, Fauci AS. Tickborne diseases–confronting a growing threat. N Engl J Med. 2018;379:701–3. 10.1056/NEJMp1807870 30044925

[6] Eldin C, Parola P. Update on tick-borne bacterial diseases in travelers. Curr Infect Dis Rep. 2018;20:17. 10.1007/s11908-018-0624-y 29789953

[7] Warren T, Lau R, Ralevski F, Rau N, Boggild AK. Fever in a visitor to Canada: a case of mistaken identity. J Clin Microbiol. 2015;53:1783–5. 10.1128/JCM.00269-15 25762775 PMC4400762

[8] Chavatte J-M, Okumura C, Landau I. Redescription of *Babesia ardeae* Toumanoff, 1940, a parasite of Ardeidae, including molecular characterization. Parasitol Res. 2017;116:1089–97. 10.1007/s00436-017-5394-1 28160075

[9] Kwak ML. Ticks in the Lion City: a preliminary review of the tick fauna of Singapore. Exp Appl Acarol. 2018;76:263–7. 10.1007/s10493-018-0305-4 30298228

[10] US Department of Health and Human Services, Food and Drug Administration. Recommendations for reducing the risk of transfusion-transmitted babesiosis: guidance for industry [cited 2020 Jan 2]. https://www.fda.gov/media/114847/download

